# Survival and adverse events of elderly patients treated with sorafenib for hepatocellular carcinoma

**DOI:** 10.3389/fonc.2022.829483

**Published:** 2022-08-02

**Authors:** Anna Soria, Mariona Calvo, Meritxell Casas, Zara Vidales, Sergio Muñoz-Martínez, Victor Sapena, Marc Puigvehi, Lidia Canillas, Raquel Guardeño, Adolfo Gallego, Beatriz Mínguez, Diana Horta, Ariadna Clos, Silvia Montoliu, Mercè Roget, Maria Reig, Mercedes Vergara

**Affiliations:** ^1^ Department of Digestive Diseases, Liver Unit, Parc Taulí University Hospital, Investigation and Innovation Institute Parc Taulí I3PT, Universitat Autònoma of Barcelona, Sabadell, Spain; ^2^ Medical Oncology Department, Catalan Institute of Oncology, Hospitalet, Barcelona, Spain; ^3^ Barcelona Clinic Liver Cancer (BCLC) Group, Liver Unit, Clinical Insitute of Digestive and Metabolic Diseases (ICMDiM), Clinic Hospital, Barcelona University, Barcelona, Spain; ^4^ Biomedical Research Center in Digestive and Liver Diseases Network (CIBERehd), Instituto Carlos III, Madrid, Spain; ^5^ Medical Statistics Core Facility, Institut d’Investigacions Biomèdiques August Pi i Sunyer (IDIBAPS), Hospital Clinic Barcelona, Barcelona, Spain; ^6^ Liver Unit, University Hospital Vall d’Hebron, Liver Diseases Research Group, Vall d’Hebron Institut of Research (VHIR), Universitat Autònoma of Barcelona, Barcelona, Spain; ^7^ Department of Digestive Diseases, Liver Unit, Hospital del Mar Medical Research Institute, Barcelona, Spain; ^8^ Medical Oncology Department, Josep Trueta University Hospital, Catalan Institute of Oncology, Girona, Spain; ^9^ Department of Digestive Diseases, Sant Pau i Santa Creu University Hospital, Barcelona, Spain; ^10^ Department of Gastroenterology, University Hospital Mútua Terrassa, Barcelona University, Terrassa, Spain; ^11^ Department of Digestive Diseases, Germans Trias i Pujol University Hospital, Badalona, Spain; ^12^ Department of Digestive Diseases, Joan XXIII University Hospital, Tarragona, Spain; ^13^ Department of Digestive Diseases, Liver Unit, Consorci Sanitari de Terrassa, Terrassa, Spain

**Keywords:** sorafenib, elderly patients, hepatocellular carcinoma, overall survival, safety, outcome

## Abstract

**Introduction:**

The first-line treatment for advanced hepatocellular carcinoma (HCC) is atezolizumab plus bevacizumab, but its availability is not universal and elderly patients are underrepresented in clinical trials. There is little evidence of efficacy and tolerability in elderly patients under systemic treatment. The aims of this study were to characterize the profile of elderly patients treated with sorafenib, assess their survival and safety profile in order to extrapolate their eligibility for systemic treatment.

**Methods:**

Retrospective multicentre study of HCC patients aged ≥75 years old treated with sorafenib from January 2008 to December 2019. Demographic data, baseline characteristics, and variables related to HCC and sorafenib were recorded. Overall survival (OS) and safety were analyzed.

**Results:**

The study included 206 patients from 11 hospitals, median age 77.9 years; 71.4% men and 62.6% stage Barcelona Clinic Liver Cancer- C (BCLC-C). The main causes of cirrhosis were hepatitis C (60.7%) and alcohol (14.7%). Most patients (84.5%) started with sorafenib 800mg and 15.5% at lower dosage. Arterial hypertension (AHT) (74.2 vs 62.2%; standardized mean differences (STD): 26) and baseline ECOG-PS>0 (45.3 vs 34.7%; STD: 38.2) differed significantly between patients receiving low and full doses. Median OS was 15.4 months (18.2 in BCLC-B vs 13.6 in BCLC-C). OS was not modified by comorbidities, age or period with more expertise.

**Conclusions:**

Sorafenib appears to be safe in elderly patients with HCC. This is the first study to characterize the profile of elderly patients to be considered for systemic treatment. These findings could be used as the reference profile for elderly candidates for atezolizumab-bevacizumab.

## Introduction

Since 2020, the treatment of choice in non-resectable hepatocellular carcinoma (HCC) has been the combination of atezolizumab and bevacizumab, which has shown better overall survival (OS) than sorafenib and has become the standard treatment for HCC (1). Previously, since 2007, sorafenib had been the standard of care in advanced HCC (2, 3). In 2018 lenvatinib was also accepted as a first-line treatment in advanced HCC owing to the results of the non-inferiority study with sorafenib (4).

Age is a crucial risk factor for the development of HCC. However, the study population in pivotal studies of sorafenib included patients with mean ages of 65 and 55 years old respectively (2, 3). Similarly, the clinical trial of atezolizumab and bevacizumab included patients with a mean age of 64 (1). In recent years, the definition of elderly people has changed; although the World Health Organisation defined the elderly as the population over 65 years old (5), due to the increase in life expectancy, most studies in this field today define the elderly as those over 75 or even 80 years old (6–9). Elderly patients frequently have more comorbidity and altered pharmacokinetics and pharmacodynamics due to polypharmacy (10). For all these reasons, physicians are more reluctant to start systemic therapy in these patients and so the clinical experience in this subgroup is limited. Management of HCC with sorafenib has received robust support from a large number of published papers since 2007. However, data in elderly patients remain scarce.

The results regarding sorafenib dosage and safety in elderly patients are controversial. Some observational studies suggest that progression-free survival, disease control and side effects are similar in the elderly and the general population (6, 7, 11–13). However, others warn that survival and safety are lower than in the general population, and that dose adjustment is frequently required (8, 9, 14, 15).

In view of this lack of consensus surrounding this subgroup of patients, the aims of this multicentre study were to assess the OS of elderly patients with HCC treated with sorafenib and to analyse the side effects and dosage adjustment needs to determine different subgroups in view to selecting patients

## Materials and methods

### Study design

Multicentre, retrospective, observational study involving a cohort of patients aged ≥75 years with HCC treated with sorafenib. The inclusion period was from January 2008 to December 2019. Eleven hospitals in Catalonia (north-eastern Spain) participated in the study.

### Study population

Applying the population age thresholds used in previous studies (6, 8), our study population included HCC patients aged ≥75 years starting sorafenib treatment. HCC was diagnosed by non-invasive criteria as determined by the American Association Study Liver Cancer (16), or by histology. None of the patients had previously received systemic therapy. Patients started sorafenib at a dosage of 400 mg, 600mg or 400 mg twice daily (800 mg daily) depending on local clinical judgement.

### Study variables

Patient data collected included demographic, medical history and baseline variables, such as features related to hepatic disease and its decompensations and cardiovascular comorbidities (AHT, diabetes mellitus, peripheral vasculopathy or ischemic cardiopathy). Regarding the HCC, baseline variables such as date of diagnosis, Barcelona Clinic Liver Cancer (BCLC) stage at the time of diagnosis and previous treatments (surgery, percutaneous ablation or trans-arterial chemoembolization) were recorded. When sorafenib was started, levels of alpha-fetoprotein and Eastern Cooperative Oncology Group (ECOG) performance status (PS) were registered. The starting date of sorafenib treatment was recorded, as were the doses administered over the course of the therapy. Sorafenib-related adverse events (AE), including hand–foot skin reaction (HFSR), diarrhoea, rash and fatigue were evaluated using the National Cancer Institute Common Terminology Criteria for Adverse Events 4.03. Early presentation of an AE was considered if it appeared during the first month of treatment. Information on whether further dose reductions were required, the date of treatment discontinuation, the cause and the start-day of the second line treatment (if administered) was recorded. Overall survival (OS) was calculated from the day of the first dose of sorafenib to the last day of follow-up or death. The time from sorafenib discontinuation to death was also assessed. In order to see whether training in sorafenib management acquired over the years might improve patient survival and follow up, we analysed OS dividing the patients into two six-year periods according to the start of sorafenib treatment: from 2008 to 2013, and from 2014 to 2019.

### Ethical considerations

The study complied with the principles of the Declaration of Helsinki. The Clinical Investigation Ethics Committee of all participating centres reviewed and approved the study with regard to the adequate fulfilment of Good Clinical Practice principles. Confidentiality was preserved in agreement with current Spanish legislation on data protection (1999). Data confidentiality was carefully preserved. Each hospital included in the study was assigned a code, which was used to identify each patient registered. The principal investigators at each centre had a separate list that was not accessible to other investigators, in which these codes were related to each patient’s clinical history number. Once the data were recorded, those clinical history numbers were dissociated from the initially assigned code. The need for informed consent was waived due to the retrospective and observational nature of the study, and because patients had advanced HCC whose treatment would have been the same regardless of whether the patients were included in the study.

### Statistical analysis

Continuous data were presented as medians and interquartile intervals [IQR: percentile 25th – 75th], and categorical data as frequencies and percentages. *Time-to-event* variables were described with median times and 95% confidence intervals (95% CI), calculated using the Kaplan-Meier method, and compared using a log-rank test. The balance between groups (initial sorafenib dosage of 400mg vs 800mg and initiation of sorafenib treatment <2013 vs ≥2013) was assessed using standardized mean differences (STD). STD >10% was considered unbalanced.

The level of significance was set at two-sided 5%. All calculations and analysis were performed with SAS 9.4 software (SAS Institute, Cary, NC, USA).

## Results

### Baseline characteristics of the study population

A total of 206 patients aged 75 or older attended at the 11 participating centres from January 2008 to December 2019 were included in the study. Baseline characteristics of the study population are represented in **Table 1**. The median age was 77.9 [IQR 76.3-80] years, and 147 (71.4%) were men. The main baseline comorbidity was AHT, recorded in 130 (63.7%), Most patients (191, 92.7%) had liver cirrhosis and the main aetiology was chronic hepatitis C, in 116 (60.7%). A group of 15 non-cirrhotic patients (7.3%), most with a diagnosis of non-alcoholic fatty liver disease, were also diagnosed with HCC. Regarding complications of cirrhosis, 46 (23%) presented detectable ascites in abdominal ultrasound (grade 1) without diuretic treatment.

**Table 1:** Patient baseline characteristics at initiation of sorafenib.

Variable	N= 206
**Age (years), median [IQR]^1^**	77.9 [76.3 - 80]
**Gender (male), n (%)**	147 (71.4)
**Cirrhosis, n (%)**	
**Yes**	191 (92.7)
**No**	15 (7.3)
**Cirrhosis aetiology, n (%)**	
**HCV^2^**	116 (60.7)
**Alcohol**	28 (14.7)
**NAFLD^3^**	18 (9.4)
**Alcohol + HCV**	13 (6.8)
**Others**	8 (4.2)
**HBV^4^**	5 (2.6)
**Alcohol + HBV**	3 (1.6)
**Comorbidities, n (%)**	
**AHT**	130 (63.7)
**Diabetes Mellitus**	78 (38.2)
**Ischemic cardiopathy**	15 (7.4)
**Peripheral vasculopathy**	8 (3.9)
**Prior history of cirrhosis decompensations**	
**Variceal haemorrhage, n (%)**	6 (3)
**Ascites, n(%)**	
**Grade I**	46 (23)
**No**	154 (77)
Prior HCC^5^ treatment, n (%)*	124 (60.2)
**Surgical resection**	20 (10.1)
**Percutaneous treatment**	54 (26.6)
**Chemoembolization**	91 (45)
**ECOG-PS^6^, n (%)**	
**0**	127 (63.8)
**1**	70 (35.2)
**2**	2 (1)
**BCLC^7^ stage, n (%)**	
**A^+^**	2 (1)
**B**	75 (36.4)
**C**	129 (62.6)
**AFP^8^ (ng/mL), median [IQR]**	17.9 [5.4 - 229]
**Bilirubin (mg/dL), median [IQR]**	1.1 [0.8 - 3.1]
**INR^9^, median [IQR]**	1.1 [1 - 1.4]
**Albumin (g/L), median [IQR]**	39.1 [36 - 42]
**Initial sorafenib dose (mg), n(%)**	
**400**	31 (15)
**600**	1 (0.5)
**800**	174 (84.5)

1. Interquartile range; 2. Chronic hepatitis C infection; 3.Non-alcoholic fatty liver disease; 4. Chronic hepatitis B infection. Missing values: Diabetes Mellitus (n=2), AHT (n=2), Ischemic cardiopathy (n=3), Peripheral vasculopathy (n=3), Variceal haemorrhage (n=5), Ascites (n=6). 5.Hepatocellular carcinoma; 6. Eastern Cooperative Oncology Group performance status; 7. Barcelona Liver Cancer Clinic; 8. Alpha-fetoprotein; 9. International Normalized Ratio. ^+^ 2 BCLC-A: 1 treatment migration, 1 not available reason, Missing values: surgical resection (n=8), percutaneous treatment (n=3), Chemoembolization (n=4), ECOG-PS (n=7).*Regarding history prior to HCC treatment, some patients received more than one alternative during follow-up.

### HCC baseline characteristics of study population

HCC baseline characteristics at the moment of initiating sorafenib are displayed in **Table 1**. The PS of 127 (63.8%) patients was 0. The BCLC stage at the moment of starting sorafenib was C in 129 patients (62.6%) and B in 75 (36.4%). One hundred and twenty-four patients (60.7%) had a history of previous HCC treatment and none had a history of liver transplant.

### Sorafenib treatment and related adverse events

The starting dose was 800mg/day in the majority of patients (84.5%); the lower dose was administered at the discretion of the physician due to renal insufficiency, AHT, intermittent coagulopathy or anticoagulant treatment. **Table 2** displays the differences in baseline characteristics between the cohorts of patients regarding starting sorafenib dose. In general, patients receiving 400mg were older and had more comorbidities (diabetes mellitus and AHT).

**Table 2:** Population baseline characteristics according to initial sorafenib dose.

Parameter	All	400mg	800mg	STD (%)^1^
**Patients, n**	205^*^	31	174	
**Age (years), median [IQR^2^]**	77.9 [76.3 - 80]	79.2 [77.5 - 80.9]	77.7 [76.2 - 79.7]	**30.2**
**Gender (male), n (%)**	146 (71.2)	21 (67.7)	125 (71.8)	8.9
**Cirrhosis, n (%)**	190 (92.7)	27 (87.1)	163 (93.7)	**22.5**
**Cirrhosis aetiology, n (%)**				**41.3**
**HCV^3^**	116 (61.1)	17 (63)	99 (60.7)	
**Alcohol**	28 (14.7)	5 (18.5)	23 (14.1)	
**NAFLD^4^**	18 (9.5)	1 (3.7)	17 (10.4)	
**Alcohol + HCV**	13 (6.8)	2 (7.4)	11 (6.7)	
**Others**	8 (4.2)	2 (7.4)	6 (3.7)	
**HBV**	4 (2.1)	0 (0)	4 (2.5)	
**Alcohol + HBV^5^**	3 (1.6)	0 (0)	3 (1.8)	
**Comorbidities, n (%)**				
**Diabetes Mellitus**	78 (38.4)	16 (51.6)	62 (36)	**31.8**
**AHT**	130 (64)	23 (74.2)	107 (62.2)	**26**
**Ischemic cardiopathy**	15 (7.4)	1 (3.2)	14 (8.2)	**21.5**
**Peripheral vasculopathy**	8 (4)	2 (6.5)	6 (3.5)	**13.6**
**Prior history of cirrhosis decompensations**				
**Variceal haemorrhage, n (%)**	6 (3)	1 (3.2)	5 (3)	1.5
**Ascites, n(%)**				
**Grade I**	46 (23.1)	1 (3.2)	45 (26.8)	**69.9**
**No**	153 (76.9)	30 (96.8)	123 (73.2)	
**Prior HCC^6^ treatment, n (%)**				
**Surgical resection**	20 (10.2)	4 (12.9)	16 (9.6)	10.3
**Radiofrequency ablation**	54 (26.7)	3 (9.7)	51 (29.8)	**52.3**
**Chemoembolization**	91 (45.3)	14 (45.2)	77 (45.3)	0.3
**ECOG-PS^7^, n (%)**				**38.2**
**0**	126 (63.6)	17 (54.8)	109 (65.3)	
**1**	70 (35.4)	12 (38.7)	58 (34.7)	
**2**	2 (1)	2 (6.5)	0 (0)	
**BCLC^8^ stage, n (%)**				**24.5**
**A**	1 (0.5)	0 (0)	1 (0.6)	
**B**	75 (36.6)	9 (29)	66 (37.9)	
**C**	129 (62.9)	22 (71)	107 (61.5)	
**Alphafetoprotein (ng/mL), median [IQR]**	17 [5.4 - 229]	19 [4.9 - 182]	17 [5.8 - 270]	**24.2**

1. Standardized mean differences; 2. Interquartile range; 3. Chronic hepatitis C infection; 4. Non-alcoholic fatty liver disease; 5. Chronic hepatitis B infection; 6. Hepatocellular carcinoma; 7. Eastern Cooperative Oncology Group performance status; 8. Barcelona Liver Cancer Clinic. Missing values: Diabetes Mellitus (n=2), AHT (n=2), Ischemic cardiopathy (n=3), Peripheral vasculopathy (n=3), Variceal haemorrhage (n=5), Ascites (n=6). Surgical resection (n=8), percutaneous treatment n=3), Chemoembolization (n=4), ECOG-PS (n=7). A patient with an initial dosage of 600mg was excluded from the analysis. The values in bold were considered unbalanced (STD>10).

The median follow-up from the initiation of sorafenib to discontinuation or death of the patient was 13.4 months [IQR 8-25]. Sorafenib safety profile is displayed in **Table 3**. During sorafenib treatment, 155 (75.2%) patients developed at least one AE, and 121 (58.7%) within the first 30 days. The most frequent AE were fatigue in 123 (59.7%). Dose adjustment was required in 103 (52.4%) patients with a median time until the first modification of 3.9 months [IQR 1.1-5.2]. Sorafenib was definitively discontinued in 182 (88.3%) patients, almost half of these (84, 46.2%) due to symptom progression. The median overall duration of sorafenib treatment was 5.6 months [IQR 1.9-12.3]: 3.4 months [IQR 1.4 - 16.8] in those with a starting dose of 400mg, and 5.9 months [IQR 2 - 12.2] in those with a starting dose of 800mg (STD 1.5, p value 0.5). During follow-up, 175 (85%) patients died and the median time from treatment discontinuation to death was 5.4 months [IQR 1.6-12.6]. Twenty-two patients (10.7%) were able to initiate second-line treatment. Comparison of treatment duration from 2008 to 2013 (5.2; [IQR 1.5 - 12]) and from 2014 to 2019 (5.6; [IQR2 - 12.9]) did not reveal any significant differences.

**Table 3:** Sorafenib treatment characteristics and adverse events.

Variable	N= 206
**Development of AE^1^, n(%)**	155 (75.2)
**HFSR^2^**	84 (40.8)
**Diarrhoea**	44 (21.4)
**Fatigue**	123 (59.7)
**Development of early AE, n(%)**	121 (58.7)
**HFSR^2^**	64 (31.1)
**Diarrhoea**	19 (9.2)
**Fatigue**	80 (38.8)
**Sorafenib treatment duration (months), median [IQR^3^]**	5.6 (1.9-12.3)
**Need for sorafenib dose adjustment, n(%)**	108 (52.4)
**Definitive sorafenib discontinuation, n(%)**	182 (88.3)
**Reason for sorafenib discontinuation, n(%)**	
**Symptomatic progression**	84 (46.2)
**Adverse events**	65 (35.7)
**Radiological progression**	23 (12.6)
**Others**	10 (5.5)
**Second-line treatment, n(%)**	22 (10.7)
**Regorafenib**	4 (18.2)
**Cabozantinib**	2 (9.1)
**Nivolumab**	5 (22.7)
**Clinical trial**	10 (45.5)
**Others**	1 (0.004)
**Death, n(%)**	175 (85)
**Time from discontinuation to death (months), median [IQR]**	5.4 (1.6-12.6)

1. Adverse event; 2. Hand-foot skin reaction; 3. Interquartile range.

The descriptive analysis of the development of AE according to initial sorafenib dose and time of occurrence is shown in **Table 4**. HSFR and diarrhoea were substantially more frequent in the 800mg group than in the 400mg group (76 [43.7%] vs 8 [25.8%] and 43 [24.7%] vs 1 [3.2%] respectively). However, fatigue was higher in the 400mg group (20 [64.5%] vs 103 [59.2%]).

**Table 4:** Adverse events according to initial dosage and time of occurrence.

Initial sorafenib dosage (mg). N (%)			
**Development of AE^1^, n (%)**		**400**	**800**
**HFSR^2^**	**No**	23 (74.2)	98 (56.3)
	**Yes**	8 (25.8)	76 (43.7)
**Diarrhoea**	**No**	30 (96.8)	131 (75.3)
	**Yes**	1 (3.2)	43 (24.7)
**Fatigue**	**No**	11 (35.5)	71 (40.8)
	**Yes**	20 (64.5)	103 (59.2)

1. Adverse events; 2. Hand-foot skin reaction. One patient started with 600mg daily and was excluded from the table.

### Overall survival and associated factors

**Figure 1:**
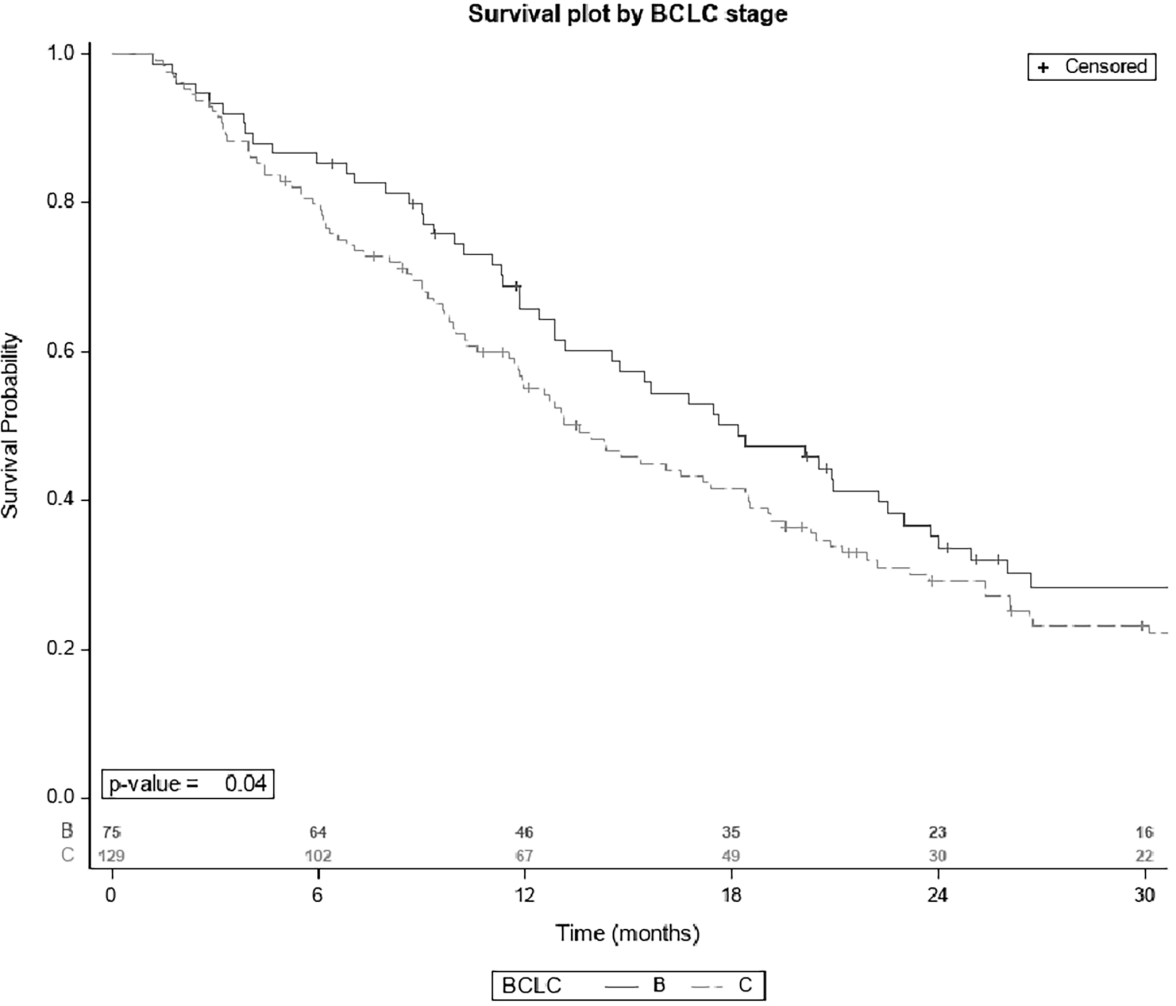
Survival plot by BCLC stage.

The median OS of the whole cohort was 15.4 months [IQR 12.9-18.4]. The univariate analysis is displayed in **Table 5**. The only prognostic factor related to OS was BCLC stage (**Figure 1**): OS was significantly longer in patients with BCLC-B stage (18.2 months [95% CI; 12.9-22.5]) than in those with BCLC-C stage (13.6 months [95% CI; 11.7-18.4]) with a p=0.04. There was no statistically significant relation between OS and comorbidities. When dividing the population into three groups according to age (≥75-80 years, >80-85 years and ≥85 years), there were no significant differences in median OS (**Table 5**).

**Table 5:** Prognostic factors related to overall survival.

**Group**	**Categories**	**Events**	**Patients at risk**	**OS^1^, months (95%CI)**	**p-value**
**All**		175	206	15.4 (12.9 - 18.4)	
**BCLC^2^**	B	63	75	18.2 (12.9 – 22.5)	0.04
	C	110	129	13.6 (11.7 – 18.4)	
**Year of treatment initiation**	<2013	60	60	13.3 (11.5 - 17.6)	0.4
	≥2013	115	146	17.4 (12.9 - 21.2)	
**AHT^3^**	No	65	74	19.6 (12.6 - 26.6)	0.06
	Yes	108	130	13.9 (11.3 - 17.4)	
**DM^4^**	No	110	126	14.8 (11.3 - 21)	0.8
	Yes	63	78	15.4 (12.7 - 19)	
**Age** (Years)	≥75 - <80	137	154	14.7 (12.6 - 19.1)	0.6
	≥80 - <85	35	48	14.5 (10.2 - 18.5)	
	≥85	3	4	23.8 (21.2 - NE)	

1. Overall survival; 2. Barcelona Liver Cancer Clinic; 3.AHT; 4. Diabetes mellitus. BCLC A patients were excluded from the analysis.

**Figure 2:**
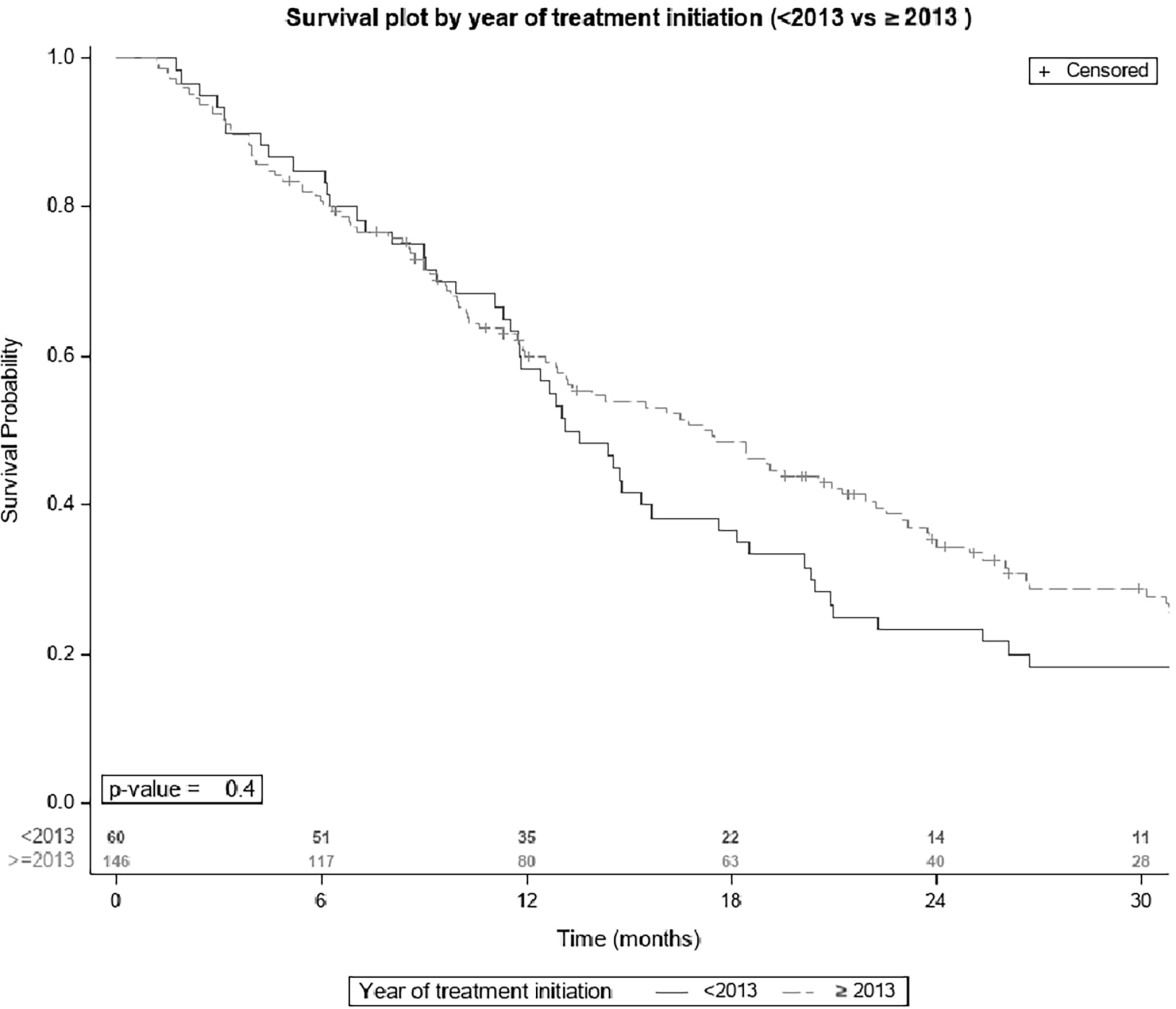
Survival plot by year of sorafenib initiation.

**Table 6** describes the differences between the basal characteristics of the cohort according to the period of treatment (2007-2013 and 2014-2019). Significant differences are found in almost all basal characteristics except for certain comorbidities (diabetes mellitus, AHT, and peripheral vasculopathy). OS in the 60 patients who started sorafenib between 2008 and 2013 was 13.3 months [IQR 11.5-17.6], and in the 115 patients who initiated this treatment after January 2013 was 17.4 months [IQR 12.9-21.2], though the differences were not significant (**Figure 2**).

**Table 6:** Population baseline characteristics at sorafenib initiation according to period of treatment.

Parameter	All	2008-2013	≥2014	STD^1^ (%)
**Patients, n**	206	60	146	
**Age (years), median [IQR^2^]**	77.9 [76.3 - 80]	77.1 [76 - 78.8]	78.2 [76.3 - 80.2]	**16.0**
**Gender (male), n (%)**	147 (71.4)	36 (60)	111 (76)	**34.9**
**Cirrhosis, n (%)**	191 (92.7)	58 (96.7)	133 (91.1)	**23.4**
**Cirrhosis aetiology, n (%)**				**70.1**
**HCV^3^**	116 (60.7)	46 (79.3)	70 (52.6)	
**Alcohol**	28 (14.7)	3 (5.2)	25 (18.8)	
**NAFLD^4^**	18 (9.4)	2 (3.4)	16 (12)	
**Alcohol + HCV**	13 (6.8)	3 (5.2)	10 (7.5)	
**Others**	8 (4.2)	0 (0)	8 (6)	
**HBV^5^**	5 (2.6)	2 (3.4)	3 (2.3)	
**Alcohol + HBV**	3 (1.6)	2 (3.4)	1 (0.8)	
**Comorbidities, n (%)**				
**AHT**	130 (63.7)	37 (63.8)	93 (63.7)	0.2
**Diabetes mellitus**	78 (38.2)	21 (36.2)	57 (39)	5.9
**Ischemic cardiopathy**	15 (7.4)	2 (3.5)	13 (8.9)	**22.5**
**Peripheral vasculopathy**	8 (3.9)	3 (5.3)	5 (3.4)	9.0
**Prior history of cirrhosis decompensations**				
**Variceal haemorrhage, n (%)**	6 (3)	3 (5.4)	3 (2.1)	**17.5**
**Ascites, n(%)**				
**Grade I**	46 (23)	17 (31.5)	29 (19.9)	**26.8**
**No**	154 (77)	37 (68.5)	117 (80.1)	
**Prior HCC^6^ treatment, n (%)**				
**Surgical resection**	20 (10.1)	4 (7.1)	16 (11.3)	**14.3**
**Radiofrequency ablation**	54 (26.6)	12 (21.1)	42 (28.8)	**17.9**
**Chemoembolization**	91 (45)	31 (54.4)	60 (41.4)	**26.3**
**ECOG-PS^7^, n (%)**				**22.6**
**0**	127 (63.8)	36 (67.9)	91 (62.3)	
**1**	70 (35.2)	17 (32.1)	53 (36.3)	
**2**	2 (1)	0 (0)	2 (1.4)	
**BCLC^8^ stage, n (%)**				**18.2**
**A**	2 (1)	0 (0)	2 (1.4)	
**B**	75 (36.4)	20 (33.3)	55 (37.7)	
**C**	129 (62.6)	40 (66.7)	89 (61)	
**Alpha-fetoprotein (ng/mL), median [IQR]**	17.9 [5.4 - 229]	44.9 [12.9 - 673]	13.9 [4.3 - 203.9]	9.3

1. Standardized mean differences; 2. Interquartile range; 3. Chronic hepatitis C infection; 4. Non-alcoholic fatty liver disease; 5. Chronic hepatitis B infection; 6. Hepatocellular carcinoma; 7. Eastern Cooperative Oncology Group performance status; 8. Barcelona Liver Cancer Clinic. Missing values: Diabetes mellitus (n=2), AHT (n=2), Ischemic cardiopathy (n=3), Peripheral vasculopathy (n=3), Variceal haemorrhage (n=5), Ascites (n=6). Surgical resection (n=8), percutaneous treatment n=3), Chemoembolization (n=4), ECOG-PS (n=7). The values in bold were considered unbalanced (STD>10).

## Discussion

A number of publications have explored the impact of age and initial sorafenib dose on outcomes in HCC patients (9, 15, 17–19). However, our study is the first to use STD analysis to establish whether there were significant baseline differences in elderly HCC patients in terms of initial sorafenib dose and treatment period that might account for the differences in OS reported in the literature.

Patient characterization is a key factor in the discussion of the treatment options for HCC. In our cohort, the patients who initiated treatment with a lower dose of sorafenib (15%) presented more comorbidities (diabetes, AHT, ischemic cardiopathy and peripheral vasculopathy) than those who started at full-dose. This information reflects the presence of two different profiles of elderly patients who may be candidates for systemic treatments. Overall, 63.7% of all elderly patients had a history of AHT but this proportion differed significantly between the groups (74.2% in low-dose and 62.2% in full-dose; STD: 26). The same pattern was observed with the ECOG-PS>0 (low-dose: 45.2% vs full-dose: 34.7%; STD: 38.2). These data show that elderly patients with a lower dose from the beginning present an unfavourable profile that may be associated with a higher incidence of AE and cirrhosis complications. An important information for the analysis would have been to have the number of medications taken by each patient. This data was taken into account when making decisions by the clinician individually in each patient. Normally the number of comorbidities is associated with the number of medications. Unfortunately, these data were not included in the database that it was created, so it cannot be analyzed individually. In the IMBRAVE trial, the rate of patients who developed AHT under sorafenib and atezolizumab-bevazizumab was 24.4 and 29.8% (for any grade) and 12.2 and 15.2% (for grade 3-4), respectively (1). Regarding the rate of fatigue in patients treated with sorafenib and atezolizumab-bevazizumab, it was 18.6 and 20.4% for any grade, and 3.2 and 2.4% for grade 3-4. Nevertheless, this trial did not consider safety as a primary or secondary end-point and elderly patients were not adequately represented, since the median age was 66 (IQR: 59–71) in the sorafenib cohort and 64 (IQR: 56–71) in the atezolizumab-bevazizumab cohort. Our study did not find any differences in terms of AE profile under sorafenib in this elderly population, but this issue must be analyzed in elderly patients treated with atezolizumab-bevazizumab where the most frequent related AE are AHT and fatigue.

In our study HSFR (43.7 and 25.8%) and diarrhoea (24.7 and 3.2%) were more frequently reported with full-dose than with low-dose sorafenib, but fatigue was more frequent in the low-dose group (64.5% vs 59.2%). However, these results should be considered as descriptive because the cohorts differed significantly in most baseline characteristics related to prognosis.

Our study did not have a control cohort of younger patients, this point being a limitation of our study. Some studies consider 70 years to consider patients as elderly. One limitation of our study is not having included patients between the ages of 70-74. Several retrospective studies (albeit with small samples) have previously ruled out differences in tolerability between groups with HCC of different ages (6, 7, 12, 13, 19, 20). Only one small retrospective study reported more AE (bleeding) in patients over 75 years old due to a greater exposure to antithrombotic drugs, and also a higher rate of treatment interruption in this subgroup due to AE (15). During the sorafenib era, there was a tendency to administer a lower starting dose in the elderly in order to avoid severe AE and complications. However, in the setting of atezo-beva dose adjustment is not possible and therefore a delay strategy has to be considered. This issue is an unmet need and should be addressed separately. Morimoto et al. (17) compared patients who initiated sorafenib treatment with half doses or standard doses. The logistic regression showed that older patients were frequently selected to receive half doses, associated with less severe AE but similar OS. In our study only 15% of patients started at half dosage so it was not possible to establish differences in OS. Dose adjustment during treatment was needed in half of the patients, mainly in the first four months. Edeline and Williet et al. support tapering off sorafenib dosage in the elderly from the beginning, especially in those >80 years, since two thirds of their patients of this age experienced grade IV AE, which in turn increased the discontinuation rate (9, 15). Tovoli et al. also found that tailoring dosage on an individualized basis in response to AE, lengthened treatment duration, increased the cumulative dose and improved OS (18). In contrast, in a cohort of 792 patients Hajiev et al. reported that a starting dose of 800mg vs 400mg a day did not modify OS in either elderly or non-elderly patients (19). So the question of whether the starting dose should be adjusted in the elderly with comorbidities from the beginning remains unanswered.

The OS in this study (15.4 months) is higher than that recorded in the largest multicentre international cohort study of patients aged 75 years or more (19), which reported an OS of 7.3 months without any differences with respect to patients below this age. The shorter survival in this international study could be due to the inclusion of an unspecified proportion of BCLC-D patients, who in our area were not administered sorafenib. In the observational studies published so far (6, 7, 12, 13, 15, 20–24) median OS differs significantly across the treatment centres in different countries, ranging from a low of 5.8 months (95% CI: 4.4-7.2) in South Korea to a high of 14.3 months in Japan (95% CI: 9.2-19.4). None of these studies found differences in OS in the elderly in comparison with the youngest, even if a higher threshold of age (80 and 85 years old) was considered (6, 7, 20). Similarly, the median OS in the present study is still higher than in the populations in those studies regardless of age. This difference is at least partially explained by the patient selection. The policy in Catalonia is more restrictive in terms of the patient’s liver function, and also because the decision to start treatment in older people may have been influenced by the general condition and frailty of the patient.

Raoul et al. (25) analysed outcomes in two different treatment periods (2007-2012 vs 2013-2017) to evaluate the impact of the physicians’ learning curve on patients’ outcomes, and found OS to be significantly longer in the second period. In the present study OS was similar in the two populations, although it was higher in the second period. Raoul et al. estimated the differences between baseline characteristics using p values rather than STD, as we did in our study; there were also significant differences between the populations in terms of BCLC stage, as they included 173 patients (92%) with BCLC-C stage while we included 129 patients (63%) with BCLC-C. Although the longer OS in both studies may reflect the incorporation of second- or third-line treatment into the HCC landscape, only 22 patients started second-line treatment in our study. Second- and third- line options were introduced in Spain in 2017, but they are still not widely available.

The main limitation of our study is its retrospective design. The physician’s subjective interpretation of the patient’s frailty is essential for deciding the dose to be administered in advanced HCC in the elderly. Using frailty scales from the beginning would help to assess all patients with HCC who are candidates for systemic treatment. In our study, the fact that the acceptance or rejection of frail patients for systemic treatment depended on the physician’s clinical judgement may have introduced a selection bias and may have been one of the reasons why the OS of our cohort was longer. These results may prompt the thought that perhaps other patients who were left untreated due to frailty might have benefited from therapy. Other limitation of the study is the lack of younger control group to analyze the results. It could be interesting to apply the same analysis of STD in this population to compare survival and adverse events. As mentioned before, we defined two groups in elderly patients depending on initial dose related to comorbidities and ECOG. Future prospective researches could be in this way in all population treated with systemic therapy.

Our study reinforces the idea that even though advanced age and comorbidity are intrinsic factors in elderly HCC, these factors should not bar elderly patients from receiving systemic treatment. Rather, elderly patients should be considered as a special population. This study has identified certain confounding factors related to the patient profile that may have a strong bearing on physicians’ decisions regarding the initiation of systemic treatment. Prospective studies in clinical practice are needed to identify predictors of tolerability, and also to address the issue of dose adjustment in this subgroup of patients.

In conclusion, this is the first study to show significant baseline differences in elderly HCC patients according to initial sorafenib dose and treatment period. It has also characterized two groups of patients on the basis of their comorbidity profile. Therefore, our data can be considered as a reference for defining the profile of elderly HCC patients who are candidates for atezolizumab-bevacizumab as first-line treatment.

## Data availability statement

The original contributions presented in the study are included in the article/supplementary material. Further inquiries can be directed to the corresponding author.

## Ethics statement

This study was reviewed and approved by Ethics comitee of Parc Tauli Sabadell. Written informed consent for participation was not required for this study in accordance with the national legislation and the institutional requirements.

## Author contributions

Garantor of the article: MV. Specific author contributions: AS Data collection, analysis and interpretation of data, draft of the manuscript and critical revision of the manuscript. MaC, MeC, ZV, SM-M, MP, LC, RG, AG, BM, DH, AC, SM: data collection and critical review of the manuscript. VS: statistical analysis, and critical revision of the manuscript. MRo: study concept and design, data collection, and critical revision of the manuscript. MRe: analysis and interpretation of data; drafting and critical revision of the manuscript, and study supervision. MV: study concept and design, data collection, analysis and interpretation of data; drafting and critical revision of the manuscript and study supervision. All authors approved the final version of the manuscript including the authorship list. All authors contributed to the article and approved the submitted version.

## Funding

AS: Travel grants from Tillots, Ferring, Norgine, Alfasigma, Jansen, Abbvie. MC: None. ZV: None. SM-M: None. VS: Travel grants from Bayer. Consultancy LEO Pharma. MP: None. LC: None. RG: None. AG: None. BM: Consultancy: Bayer-Shering Pharma, Eisai-Merck. Conferences/lectures: Eisai, MSD, Roche. Research grant: Lab Viñas. Funding: BM is funded by grants PI18/00961 and PI21/00714 from Instituto de Salud Carlos III. DH. None. AC. None. SM. Conferences/lectures: Bayer. Travel grants: Bayer, and Eisai. MRo. None. MRe. Consultancy: Bayer-Shering Pharma, BMS, Roche, Ipsen, AstraZeneca, Lilly. BTG/Paid conferences: Bayer-Shering Pharma, BMS, Gilead, Lilly. Research Grants: Bayer-Shering Pharma, Ipsen. MV. Travel grants: Gilead, MSD, Bayer, Abvie. Conferences/lectures: MSD, Gilead, Abvie, Eisai.

## Acknowledgments

We sincerely thank Mireia Miquel, Jorge Sánchez-Delgado, Cristina Solé and Montserrat Gil from Parc Taulí Hospital; Álvaro Díaz, Marco Sanduzzi-Samparelli, Neus Llarch and Gemma Iserte from Hospital Clínic, Barcelona; Berta Laquente, Maica Galán and Eduard Fort from the Institut Català d’Oncologia-L’Hospitalet; Margarita Sala from Josep Trueta Hospital; Susana Coll from the Hospital del Mar Medical Research Institute; Agnes Raga from Mutua Terrassa Hospital and finally Jordi Ortiz from Terrassa Consorci Hospital for their contributions to the study. We sincerely thank Michael Maudsley for their English correction.

## Conflict of interest

The authors declare that the research was conducted in the absence of any commercial or financial relationships that could be construed as a potential conflict of interest.

## Publisher’s note

All claims expressed in this article are solely those of the authors and do not necessarily represent those of their affiliated organizations, or those of the publisher, the editors and the reviewers. Any product that may be evaluated in this article, or claim that may be made by its manufacturer, is not guaranteed or endorsed by the publisher.

## Abbreviations

HCC, hepatocellular carcinoma; BCLC, Barcelona Clinic Liver Cancer; ECOG, Eastern Cooperative Oncology Group; PS, performance Status; AE, Adverse Events; HFSR, hand food skin reactions; OS, Overall survival; STD, standardized mean differences.
